# Prioritizing cardiovascular disease-associated variants altering NKX2-5 and TBX5 binding through an integrative computational approach

**DOI:** 10.1016/j.jbc.2023.105423

**Published:** 2023-11-04

**Authors:** Edwin G. Peña-Martínez, Diego A. Pomales-Matos, Alejandro Rivera-Madera, Jean L. Messon-Bird, Joshua G. Medina-Feliciano, Leandro Sanabria-Alberto, Adriana C. Barreiro-Rosario, Jeancarlos Rivera-Del Valle, Jessica M. Rodríguez-Ríos, José A. Rodríguez-Martínez

**Affiliations:** 1 Department of Biology, University of Puerto Rico Río Piedras Campus, San Juan, Puerto Rico; 2 Department of Biology, University of Puerto Rico Cayey Campus, Cayey, Puerto Rico

**Keywords:** transcription factors, single-nucleotide polymorphism (SNP), gene regulation, cardiovascular disease, computational biology, DNA-binding protein, genomics

## Abstract

Cardiovascular diseases (CVDs) are the leading cause of death worldwide and are heavily influenced by genetic factors. Genome-wide association studies have mapped >90% of CVD-associated variants within the noncoding genome, which can alter the function of regulatory proteins, such as transcription factors (TFs). However, due to the overwhelming number of single-nucleotide polymorphisms (SNPs) (>500,000) in genome-wide association studies, prioritizing variants for *in vitro* analysis remains challenging. In this work, we implemented a computational approach that considers support vector machine (SVM)-based TF binding site classification and cardiac expression quantitative trait loci (eQTL) analysis to identify and prioritize potential CVD-causing SNPs. We identified 1535 CVD-associated SNPs within TF footprints and putative cardiac enhancers plus 14,218 variants in linkage disequilibrium with genotype-dependent gene expression in cardiac tissues. Using ChIP-seq data from two cardiac TFs (NKX2-5 and TBX5) in human-induced pluripotent stem cell-derived cardiomyocytes, we trained a large-scale gapped k-mer SVM model to identify CVD-associated SNPs that altered NKX2-5 and TBX5 binding. The model was tested by scoring human heart TF genomic footprints within putative enhancers and measuring *in vitro* binding through electrophoretic mobility shift assay. Five variants predicted to alter NKX2-5 (rs59310144, rs6715570, and rs61872084) and TBX5 (rs7612445 and rs7790964) binding were prioritized for *in vitro* validation based on the magnitude of the predicted change in binding and are in cardiac tissue eQTLs. All five variants altered NKX2-5 and TBX5 DNA binding. We present a bioinformatic approach that considers tissue-specific eQTL analysis and SVM-based TF binding site classification to prioritize CVD-associated variants for *in vitro* analysis.

Cardiovascular diseases (CVDs) are the leading cause of death worldwide and encompass multiple disorders (coronary artery disease, congenital heart disease, stroke, etc.), many of which are heritable (1, 2, 3, 4, 5). Genome-wide associations studies (GWAS) have mapped over 90% of CVD-associated variants within noncoding regions of the genome (6, 7). Noncoding single nucleotide polymorphisms (SNPs) can impact phenotype by altering gene regulatory mechanisms, such as transcription factor (TF)-DNA binding and gene expression (8, 9, 10, 11). NKX2-5 and TBX5 are cardiac TFs that regulate gene expression in the developing heart (12, 13, 14, 15, 16, 17). Previous research has identified CVD-associated SNPs that alter cardiac TF-DNA binding, but further research is required to establish causality (18, 19, 20, 21, 22). However, with the overwhelming number of GWAS SNPs (>500,000), prioritizing candidate CVD-causing variants for experimental validation remains challenging.

One approach to address this challenge is the integration of functional genomic datasets with predictive models to identify variants that create or disrupt TF binding sites (TFBSs) (23, 24, 25, 26). Large-scale gapped k-mer (LS-GKM) support vector machine (SVM) models can be trained to predict TFBSs by using *in vitro* genome-wide binding data from chromatin immunoprecipitation followed by DNA sequencing (ChIP-seq). LS-GKM-SVM models have several advantages over traditional position weight matrix (PWM)-based methods, by considering complex sequence features like dinucleotide interactions, longer/gapped k-mers, and intracellular patterns (27, 28, 29, 30). LS-GKM-SVM models can be trained with ChIP-seq data from specific cell lines or tissue to integrate relevant epigenomic and regulatory context (23).

In this work, we prioritize noncoding variants associated with CVDs and CV traits using public data from the GWAS catalog (31), genome tissue expression (GTEx) portal (32), ENCODE (33), ChIP-Atlas (34), and Remap (35). We compiled a list of CVD-associated SNPs linked with a genotype-dependent gene expression in cardiac tissue and trained a LS-GKM-SVM model with ChIP-seq data from NKX2-5 and TBX5 in human-induced PSC-derived cardiomyocytes (HiPSC-CMs). Both models were used to score previously identified heart DNase I hypersensitivity genomic footprints (DGFs) (36) that colocalize within putative cardiac enhancers (37) and tested them through *in vitro* binding by electrophoretic mobility shift assay (EMSA). Our predictive model was successful at identifying NKX2-5 and TBX5 binding sites and distinguishing between DNA sequences with different binding affinities.

Having validated DGF scored by the GKM-SVM model, we calculated the impact of CVD-associated SNPs in NKX2-5 and TBX5 binding. Five variants (rs59310144, rs6715570, rs61872084, rs7612445, and rs7790964) predicted to alter NKX2-5 and TBX5 binding were prioritized for *in vitro* validation based on the predicted change in binding and expression quantitative trait loci (eQTLs) in cardiac tissue. The five variants were validated through EMSA and resulted in changes in NKX2-5 and TBX5 DNA binding. In short, we present a bioinformatic approach that considers tissue-specific eQTL analysis and SVM-based TFBS classification to prioritize functional CVD-associated SNPs.

## Results

### Identification of CVD-associated SNPs in cardiac eQTL

**Figure 1:**
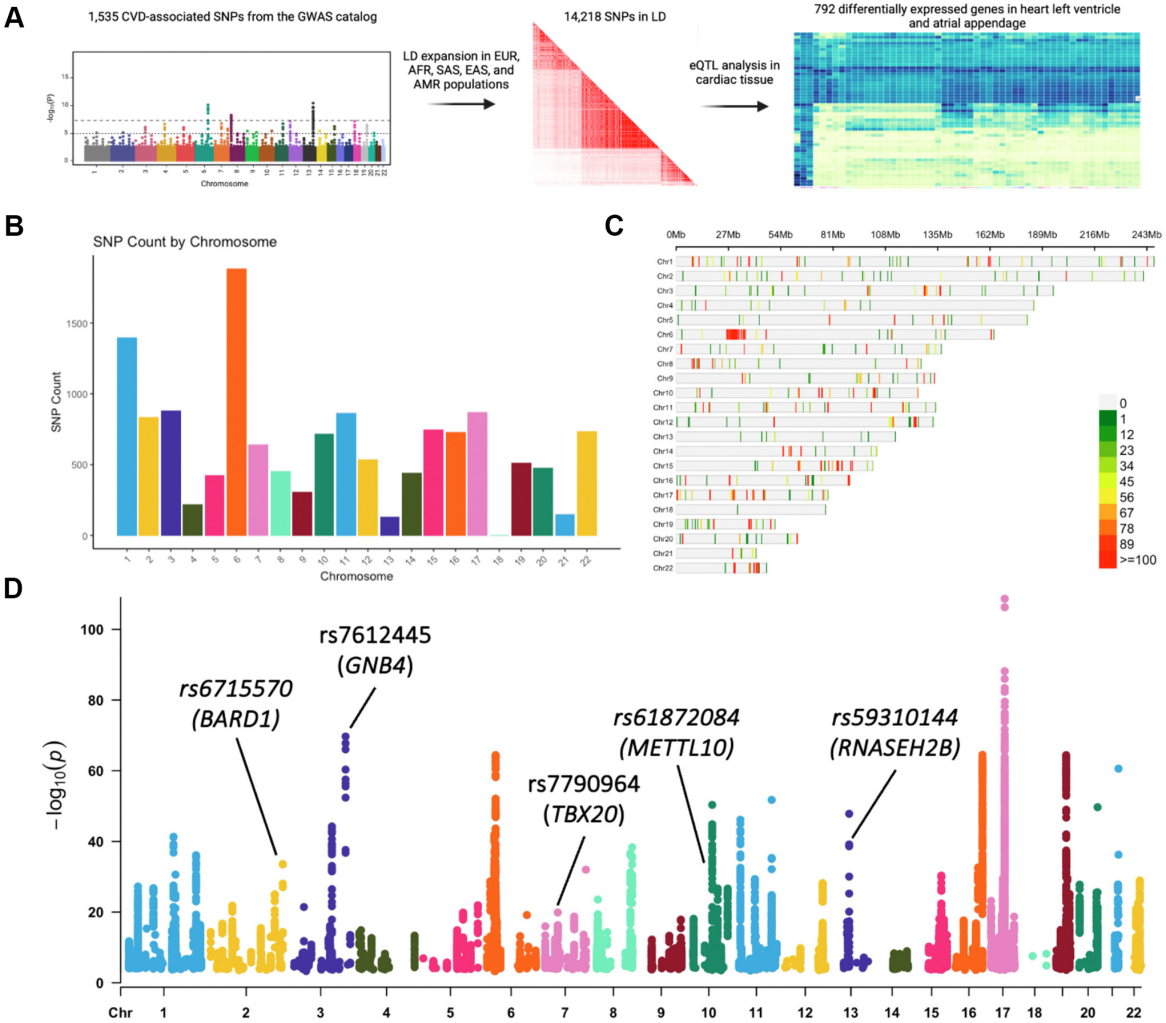
**Identificationof CVD-associated SNPs**. *A*, pipeline to identify potential CVD-causing SNPs. *B*, number of CVD-associated SNPs per chromosome. *C*, distribution of SNP frequency within autosomal chromosome, binned by 1 Mb windows. *D*, SNP-gene pairs with genotype-dependent expression in cardiac tissue. Each dot represents a SNP-gene pair with genotype-dependent expression in the heart atrial appendage or left ventricle in one or more populations. rs6715570-BARD1, rs61872084-METTL10, rs59310144-RNASEH2B, rs7612445-GNB4, and rs7790964-TBX20 are SNP-gene pairs that were evaluated *in vitro* in Figure 3. CVD, cardiovascular disease; LD, linkage disequilibrium; SNP, single nucleotide polymorphism.

To identify potential CVD-causing SNPs, we downloaded the GWAS catalog and filtered the data to keep cardiovascular disease or trait-associated SNPs (*e.g.*, congenital heart defects, cardiomyocyte differentiation, stroke, arrhythmia, etc.; full list of SNPs in File S1). We then intersected the CVD-associated SNPs with a catalog of putative fetal and adult heart enhancers and genomic footprints of fetal hearts, resulting in 1535 genomic variants. The CVD-associated SNP set was expanded to include SNPs in linkage disequilibrium (LD r^2^ > 0.8) from diverse populations (EUR, AFR, SAS, EAS, and AMR) and resulted in 14,218 unique SNPs occurring in one or more populations. To evaluate the potential of these SNPs to be biologically relevant in cardiovascular biology, we analyzed gene expression patterns in cardiac tissue with the previously identified variants in the GTEx portal. We found 792 genes with genotype-dependent activity in the heart atrial appendage or left ventricle associated with the previously identified SNPs. The workflow is illustrated in Fig. 1*A*, and the list of SNPs associated with differentially expressed genes in cardiac tissue is in File S1. We identified chromosomes with a higher frequency of CVD-associated SNPs, with >1000 variants (chromosomes 1 and 6) and ∼500 (chromosomes 2, 3, 7, 10, 11, 12, 15, 16, 17, 19, and 22), including those in LD (Fig. 1*B*). Chromosomes with a high SNP frequency may have variants evenly distributed among them, like chromosomes 1 and 2, while others contain multiple variants in the same (or near) loci, like chromosomes 6, 10, 15, and 22 (Fig. 1*C*). We also analyzed data from the GTEx database and found genes with genotype-dependent expression in cardiac tissue (heart atrial appendage and left ventricle) containing the identified CVD-associated SNPs or the variants in LD. We identified 31,122 SNP-gene pairs (792 unique genes) with genotype-dependent expression in cardiac tissue (Fig. 1*D*).

### Training LS-GKM SVM model to score NKX2-5 and TBX5 binding sites

**Figure 2:**
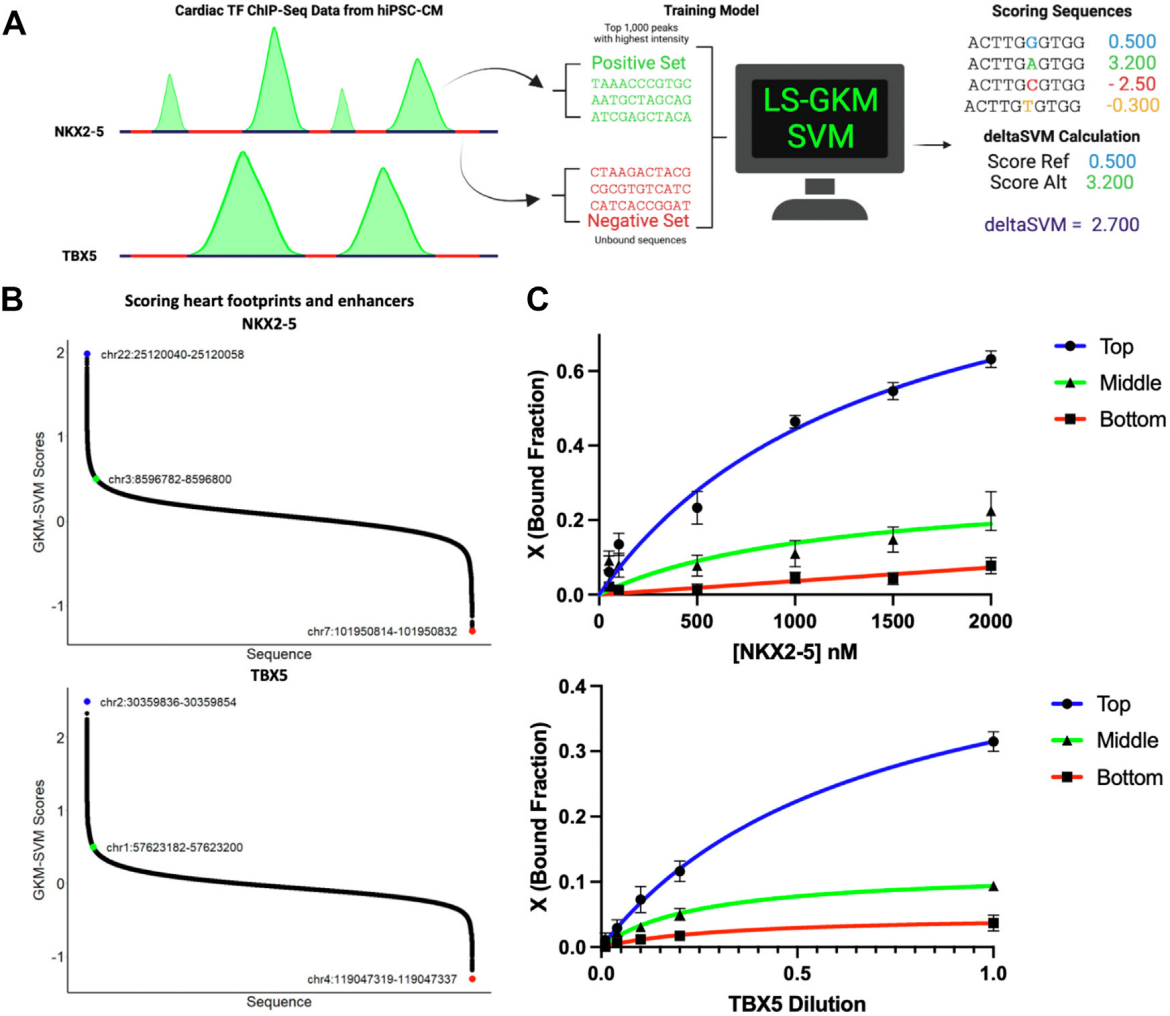
**Training and testing of LS-GKM SVM predictive model**. *A*, schematic of model training with NKX2-5 and TBX5 ChIP-seq data from HiPSC-CM. *B*, scoring of ∼520,000 DGF that occur in heart enhancers with the NKX2-5 (*top*) and TBX5 (*bottom*) predictive models. *C*, *in vitro* testing of predictive model for highest, middle, and lowest scored sequences for NKX2-5 (*top*) and TBX5 (*bottom*). For NKX2-5, we tested chr22:25,120,040 to 25,120,058 (*circle* with *blue line*), chr3:8,596,782 to 8,596,800 (*triangle* with *green line*), and chr7:101,950,814 to 101,950,832 (*square* with *red line*). For TBX5, we tested chr2:30,359,836 to 30,359,854 (*circle* with *blue lines*), chr1:57,623,182 to 57,623,200 (*triangle* with *green line*), and chr4:119,047,319 to 119,047,337 (*square* with *red line*). HiPSC-CM, human-induced PSC-derived cardiomyocytes; LS-GKM, large-scale gapped k-mer; SVM, support vector machine; TF, transcription factor.

We trained a LS-GKM SVM model to prioritize CVD-associated SNPs that alter DNA binding by TFs known to play important roles in heart development and biology. The models were trained using NKX2-5 and TBX5 ChIP-seq data from HiPSC-CMs. The 1000 top-scoring ChIP-seq peaks were used as a positive training set, while unbound sequences of the same length, GC content, and chromosome were used as negative training (Fig. 2*A*) The best-performing LS-GKM SVM classifier model trained with NKX2-5 ChIP-seq data (SRX9284027) (38) obtained an AUROC value of 0.955 and an AUPRC value of 0.954. The best TBX5 (SRX2023721) (39) model obtained an AUROC value of 0.921 and an AUPRC value of 0.912 (Fig. S1, *A* and *B*). The models were used to score all possible 2,097,152 nonredundant 11 bp oligomers (11-mers). The 11-mer scores were sorted, and the 1000 top-scoring sequences were used to generate a PWM using multiple Em for motif elicitation and observed similar motifs to those previously reported for NKX2-5 (40, 41) and TBX5 (42) (Fig. S1, *C* and *D*). We proceeded to score ∼520,000 fetal heart DGF that occur heart enhancers to identify genomic loci potentially bound by NKX2-5 or TBX5 (Fig. 2*B*). We then chose the DNA sequences with the highest, middle, and lowest scores to test for *in vitro* binding through EMSA (Figs. 2*C* and S2). As predicted by the GKM SVM model, sequences with a higher score resulted in increased NKX2-5 and TBX5-DNA binding.

### CVD-associated SNPs alter NKX2-5 and TBX5-DNA binding

**Figure 3:**
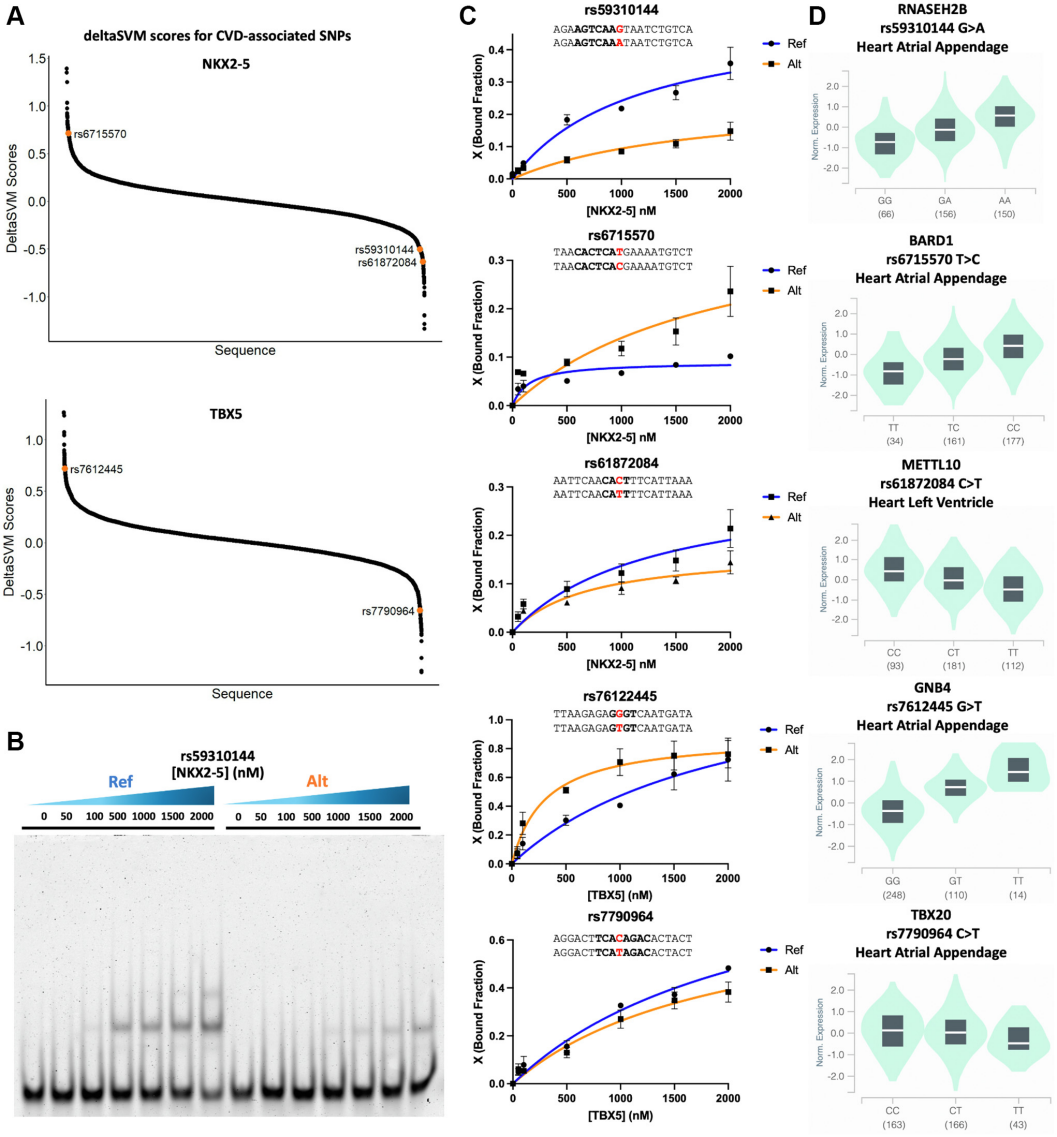
**CVD-associated SNPs alter NKX2-5 and TBX5 *in vitro* binding**. *A*, DelstaSVM score distribution of the 14,218 CVD-associated SNPs for NKX2-5 (*top*) and TBX5 (*bottom*). *B*, representative EMSA gel for rs59310144 reference/noneffect (Ref) and alternate/effect (Alt) alleles. Source image is available in Fig. S3. *C*, binding curves of rs59310144, rs6715570, rs61872084, rs76122445, and rs7790964. Experiments were performed in triplicates and binding curves show average bound fraction (X) and error bars are standard error. *D*, cardiac tissue eQTL analysis of *RNASEH2B*, *BARD1*, *METTL10*, *GNB4*, and *TBX20* expressed in heart atrial appendage or left ventricle when rs59310144, rs6715570, rs61872084, rs76122445, and rs7790964 occur, respectively. CVD, cardiovascular disease; SNP, single nucleotide polymorphism.

After successful training and validation of the LS-GKM SVM predictive model, we proceeded to score the 14,218 SNPs to prioritize variants that may directly contribute to CVDs. Both reference and alternate allele sequences were scored to predict fold change (deltaSVM score) of TF-DNA binding. We selected five SNPs (rs59310144, rs6715570, rs61872084, rs7612445, and rs7790964) that deltaSVM predicted significant change in NKX2-5 or TBX5 binding and are associated with genotype-dependent expression in cardiac tissue (Fig. 3*A* and Table S2). When evaluated through EMSA, we observed a difference in NKX2-5 and TBX5 DNA binding between reference and alternate for all five SNPs (Figs. 3, *B* and *C* and S3). Variants rs59310144 and rs61872084 resulted in a decrease in NKX2-5 DNA binding, while rs6715570 increased binding. Variant rs7612445 resulted in an increase in TBX5 binding, while rs7790964 decreased binding. We found that all five SNPs were in eQTLs described in cardiac tissue and identified five genes with genotype-dependent expression in the heart atrial appendage or left ventricle (Fig. 3*D*).

## Discussion

As we continue to research the genetic basis for human disease, the number of identified functional/causal noncoding SNPs continues to grow. Understanding and prioritizing SNPs that contribute to the disease phenotypes is essential. However, there is a lack of consensus or bioinformatic protocol to prioritize noncoding SNPs that are biologically relevant in the development of human diseases (25). To address this challenge, we applied a GKM-SVM–based model to identify and prioritize potential CVD-causing variants for experimental validation. We leveraged on public data from the GWAS catalog and extracted SNPs that were associated with cardiovascular disease or traits and included variants in LD from multiple populations (EUR, AFR, SAS, EAS, and AMR). The distribution of the identified CVD-associated SNPs was not uniform throughout the genome (Fig. 1, *B* and *C*). This suggests that certain chromosomes, or specific loci, are enriched with CVD-associated SNPs and may contribute to the cardiac phenotype. SNPs in eQTL were identified to prioritize variants with genotype-dependent expression in cardiac tissue (left ventricle and atrial appendage). Through this approach, we aimed to narrow the extensive list of noncoding variants and identify SNPs that may contribute to CVDs.

To further prioritize variants that may contribute to a cardiac phenotype, we trained a GKM-SVM classifier to identify SNPs that disrupt NKX2-5 and TBX5-DNA binding. Because TFs regulate gene expression in a tissue-specific manner (43, 44), we trained the predictive model with ChIP-seq data from NKX2-5 and TBX5 collected in HIPSC-CM. After testing the model by scoring all possible 11-mers, we generated PWMs for both models resulting in DNA binding motifs in agreement with previously described motifs for NKX2-5 and TBX5 (Fig. S1) (40, 41, 42). The GKM-SVM model was successful at identifying binding sites within heart DGF for both NKX2-5 DBD and full-length TBX5 (Fig. 2, *B* and *C*). Our results suggest that the LS-GKM SVM model will be able to successfully predict changes in binding affinity between reference and variant DNA sequences that alter cardiac TF-DNA binding. Functional genomics studies have identified millions of putative enhancers, but the identity of most bound TFs is unknown (33). Our study can contribute to identifying transcriptional regulators active in specific tissues or environments.

We tested five SNPs that altered NKX2-5- (rs59310144, rs6715570, and rs61872084) and TBX5-(rs7612445 and rs7790964) DNA binding activity and associated with genotype-dependent expression (*RNASEH2B*, *BARD1*, *METTL10*, *GNB4*, and *TBX20*, respectively) in cardiac tissue (Fig. 3, *C* and *D*). Four of the variants (rs6715570, rs61872084, rs76122445, and rs7790964) effect allele’s impact on gene expression were proportional to the changes in TF-DNA binding activity. Inversely, the effect allele of variant rs59310144 increased gene expression, while TF-DNA binding decreased. *RNASEH2B*, *BARD1*, *GNB4*, and *TBX20* have been previously identified to be differentially expressed in the heart atrial appendage when variants rs59310144, rs6715570, rs7612445, and rs7790964 (respectively) occur. *RNASEH2B*, which has been previously found to be differentially expressed in CVD risk events, is upregulated when the alternate allele of variant rs59310144 is present (45). NKX2-5 can act as either an activator or repressor (46, 47, 48). Benaglio *et al*. (2019) used a combination of EMSA, luciferase reporter assays, and CRISPRi to describe how the stronger TF-binding allele (rs590041) was associated with reduced *SSBP3* expression. In contrast, the stronger TF binding allele (rs3807989) increased CAV1 and CAV2 expression (19). This suggests that disruption of the NKX2-5 binding site by rs59310144 could increase *RNASEH2B* expression. *BARD1* has also been identified as upregulated when the alternate allele of variant rs6715570 occurs in the heart atrial appendage. Copy number alterations in the *BARD1* locus have been associated with developmental delays, including coarctation of the aorta during early organogenesis and heart development (49). Variant rs61872084 has been identified in the heart's left ventricle when *METTL10* (methyltransferase-like protein 10) is downregulated when the alternate allele occurs. Accumulation of METTL10 methylated products, such as S-adenosyl-L-methionine, S-adenosyl-L-homocysteine, and homocysteine have been correlated with kidney dysfunction and CVDs in patients with type 2 diabetes (50). G protein subunit beta 4 (GNB4) has been previously associated with the regulation of heart rate stability and identified in patients with higher cardiovascular mortality risk (51). TBX20, a cardiac TF regulated by TBX5, is essential for proper heart development with mutations that have been associated with congenital heart diseases (52).

Our findings suggest that NKX2-5 regulation of the *RNASEH2B* (repression), *BARD1* (activation), and *METTL10* (activation), and TBX5 regulation of *GNB4* (activation) and *TBX20* (activation), genes are possible mechanisms that can be further explored to establish rs59310144, rs6715570, rs61872084, rs76122445, and rs7790964 as causal CVD risk-variants. Although the etiology of human diseases is complex and multifactorial, this approach can provide crucial information that can be implemented during *in vivo* experiments or clinical research to address genetic diseases caused by noncoding SNPs. In summary, we believe this bioinformatic approach, which considers tissue-specific eQTL analysis and SVM-based TFBS classification, is a scalable method that can be applied to multiple types of human diseases.

## Experimental procedures

### Data

ChIP-seq data sets for NKX2-5 and TBX5 from HiPSC-CMs were collected from the ChIP-Atlas (34) and Remap (35) databases. DNase I hypersensitivity footprints for fetal heart tissue (left atrium, right ventricle), heart fibroblast, and differentiated cardiomyocytes were obtained from ENCODE (ENCSR764UYH) (33). Putative heart enhancers were downloaded from the supplementary files from Dickel *et al*. (37) Disease or trait-associated SNPs were downloaded from the GWAS catalog (gwas_catalog_v1.0-associations_e0_r2022–11–29.tsv).

### Model training

LS-GKM was implemented to perform predictions on TF-DNA binding affinity for NKX2-5 and TBX5 (53, 54). LS-GKM was downloaded through the Comprehensive R Archive Network for Linux, Mac OS, and Windows platforms. For each TF ChIP-seq bed file, peaks were sorted by intensity, and the top 1000 peaks were used as a positive set for training the models. The *genNullSeqs* function from the gkmSVM package in R was used to generate negative training by selecting unbound sequences of the same length, chromosome, and GC content as the positive training file. The *gkmtrain* function was used to train the SVM classifiers. The following parameters were used to train the model using a 5-fold cross-validation: word length (*l*) = 11 and the number of informative positions (*k*) = 7 (gkmtrain -x 5 -L 11 -k 7 -d 3 -C 1 -t 2 -e 0.005). Model performance was assessed *via* receiver operator characteristic and precision-recall curves area under the curve using the gkmSVM package in R.

### Sequence scoring

The models for each TF were used to predict TF-DNA binding through weighted scoring. The gkmpredict function was used to score 18 bp sequences within 519,540 DGF from cardiac tissue that were found within previously identified human heart enhancers. These sequences were identified by intersecting genomic coordinates of ∼1.6 million DGFs from cardiac tissue with ∼80,000 putative enhancers active in fetal and adult human hearts (36, 37). Parameters were set to their default values, and *gkmpredict* was used to generate an output file listing all sequences and their respective assigned scores by the classifier model for NKX2-5 and TBX5 binding predictions. Positive scores predicted TF-DNA binding, while negative scores predicted no binding activity.

### Position weight matrix from LS-GKM models

We scored and sorted every possible 11-mers and selected the top 1000 sequences for the generation of a PWM using the multiple Em for motif elicitation (55) web-based tool default parameters to generate a logo.

### Cardiovascular disease-associated risk-variants identification

Variants from the GWAS catalog (gwas_catalog_v1.0-associations_e0_r2022–11–29.tsv) were downloaded and filtered to identify CVD or trait-associated SNPs. Insertions, deletions, and incomplete entries from the GWAS catalog were removed. Variants were filtered from the “DISEASE/TRAIT” column using the following function:

*grepl(‘heart|cardiac|aortic|atrial|ventric|cardio|vascular|artery|coronary|myocardial|valve|cardio|cardium|stroke'*, `*DISEASE/TRAIT*`)

CVD SNPs were intersected with human putative enhancers active in the human heart and DGF from the fetal heart. CVD-associated SNPs that occur within human heart enhancers and footprints were expanded to include variants in LD using the LDLinkR package (56). CVD-associated SNPs and variants in LD with genotype-dependent expression in cardiac tissue (heart atrial appendage and left ventricle) were identified through the GTEx Portal database. The GTEx portal reports permutation-adjusted *p*-values for each gene for the most significant SNP per gene. The *r2d_threshold* was set to >0.8, and the p*_threshold* argument was left at default (*p*-value < 0.1).

### NKX2-5 and TBX5 expression and purification

The NKX2-5 homeodomain gene (Asp16 to Leu96) was cloned in pET-51(+) expression vector containing an N-terminal Strep•Tag II and a C-terminal 10 × His•Tag through Gibson Cloning and purified through Ni-NTA affinity chromatography, as previously described (18). The TBX5 T-box domain gene (Met51 to Ser248) was cloned into a pET expression vector with a 6× His•Tag (VectorBuilder Inc) and purified through Ni-NTA affinity chromatography, as previously described (18) (Fig. S4*A*). The human TBX5 gene (Clone ID HsCD00079979, DNASU Plasmid Repository) was cloned in pEU-E01-GST-TEV-MCS-N1 (Cambridge Isotope Laboratories, Inc CFS-PEU-V1.0) vectors using Gibson Assembly (New England Biolabs, Inc). Clones were verified by Sanger Sequencing from the University of Wisconsin Biotechnology Center DNA Sequencing Facility. TBX5 was expressed using the Wheat Germ Cell-Free Protein Expression from the CellFree Sciences Co following the manufacturer’s protocol. Protein expression was confirmed through SDS-PAGE followed by Western Blot using Anti-GST HRP-conjugated (dilution: 1:10,000) NB100–63173) antibody (Novus Biological) (Fig. S4*B*).

### Electrophoretic mobility shift assay

NKX2-5 and TBX5 binding were evaluated using 20 bp genomic sequences centered on the candidate SNP plus 20 bp constant sequence for primer binding and extension. The primer contained IR-700 fluorophore. All oligonucleotides were purchased from Integrated DNA Technologies, Inc and sequences are in Table S1. The IR-700 fluorophore was added through a primer extension reaction and purified using EconoTaq Plus (Lucigen, 30,035–2), the QIAquick PCR Purification Kit (Qiagen 28,106). Twenty microliter binding reactions were performed in binding buffer (50 mM NaCl, 10 mM Tris-HCl (pH 8.0), and 10% glycerol) and 5 nM fluorescently labeled dsDNA. Five concentration points were used for purified NKX2-5 homeodomain and TBX5 T-box ranging from 50 nM to 2000 nM. Cell-free expressed TBX5-DNA binding was evaluated using four TBX5 dilutions (1, one-fifth, 1/10, and 1/25) of the cell-free extract. Binding reactions were incubated for 30 min at 30 °C followed by 30 min at room temperature before loading onto a 6% polyacrylamide gel in 0.5× TBE (89 mM Tris/89 mM boric acid/2 mM EDTA, pH 8.4). The gel was pre-ran at 85 V for 15 min, loaded at 30 V, and resolved at 75 V for 1.5 h at 4 °C. Gels were imaged with Azure Sapphire Bio-molecular Imager with 658 nm excitation and 710 nm emission.

Binding curves were generated by first quantifying the fluorescence signal in each DNA band using ImageJ (57). Background intensities obtained from blank regions of the gel were subtracted from the band intensities. The fraction of bound DNA was determined using Equation 1. The fraction of bound DNA was plotted *versus* the TF concentration. Binding curves were obtained by “one-site–specific binding” nonlinear regression using Prism software.

Equation 1. Binding affinity from the integrated density of bound and unbound bands.

$$Fraction\;bound=\frac{bound}{\left(bound+unbound\right)}$$

## Data availability

All data and supplementary material generated for this study are publicly available at https://github.com/joshuagmedina/cardioDisease_riskVariants.

## Supporting information

This article contains supporting information.

## Conflict of interest

The authors declare no conflicts of interest with the contents of this article.

## Supporting information

Supplemental File 1

Supplementary Figure S1 Performance parameters and motif analysis. *A* and *B*, performance parameters of (*A*) NKX2-5 and (*B*) TBX5 as determined by their receiver operating characteristics (ROC) and precision-recall curves. *C* and *D*, binding motif for (*C*) NKX2-5 and (*D*) TBX5 after scoring all possible 11-mers and generating a PWM logo. PWM, position weight matrix.

Supplementary Figure S2 EMSA analysis of heart footprint and enhancers for NKX2-5 (*left*) and TBX5 (*right*). All EMSA were performed and triplicates. EMSA, electrophoretic mobility shift assay.

Supplementary Figure S3 EMSA analysis of five CVD-associated SNPs. Figure 3*B* was derived from EMSA gel of rs59310144 (*top left*). EMSA, electrophoretic mobility shift assay; CVD, cardiovascular disease; SNP, single nucleotide polymorphism.

Supplemental Figure S4 legend

Supplementary Tables Supplementary Table 1: Oligonucleotides used in this work. Supplementary Table 2: CVD-associated SNPs with differential gene expression in cardiac tissue and predicted impact on NKX2-5 DNA binding.
