# Supplementary figures and images for: Prioritizing cardiovascular disease-associated variants altering NKX2-5 and TBX5 binding through an integrative computational approach
Source: J Biol Chem. 2023 Nov 4;299(12):105423. doi: 10.1016/j.jbc.2023.105423 (PMC10750078; doi:10.1016/j.jbc.2023.105423)

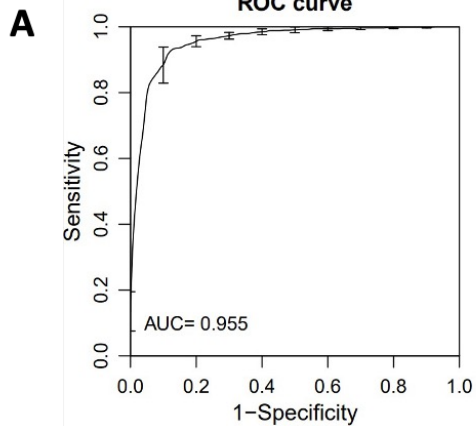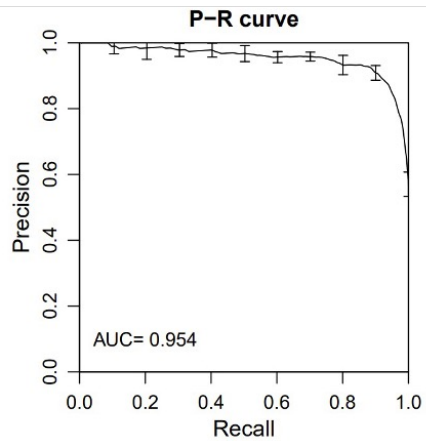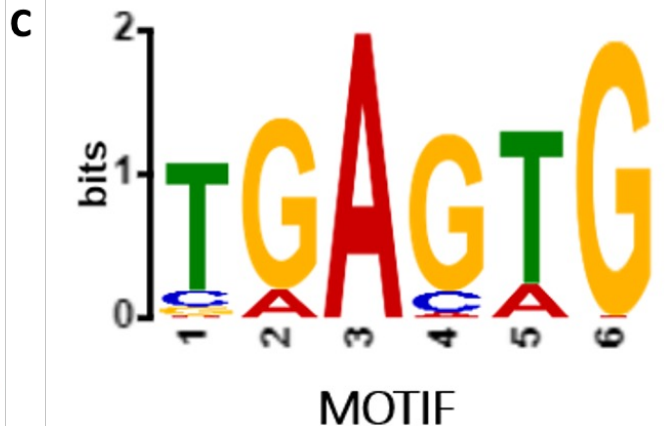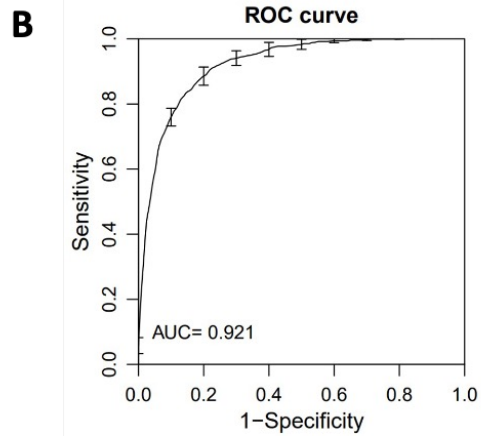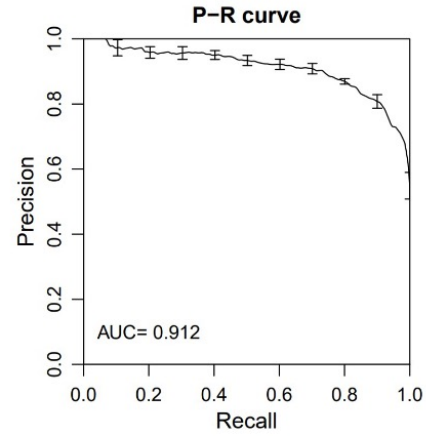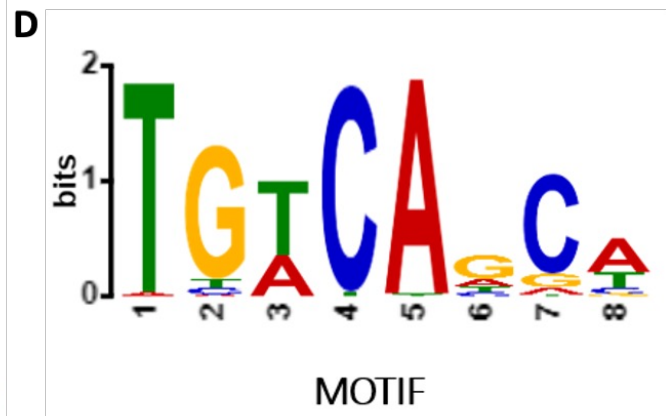

Supplement: Supplementary Figure S1 — Performance parameters and motif analysis. A and B, performance parameters of (A) NKX2-5 and (B) TBX5 as determined by their receiver operating characteristics (ROC) and precision-recall curves. C and D, binding motif for (C) NKX2-5 and (D) TBX5 after scoring all possible 11-mers and generating a PWM logo. PWM, position weight matrix. [file mmc2.pdf]

chr22:25120040-25120058

[NKX2-5] (nM)

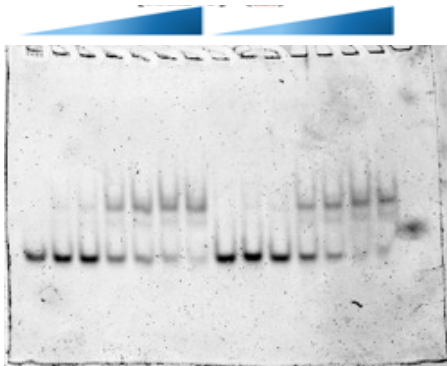

chr2:30359836-30359854

TBX5 (μL)

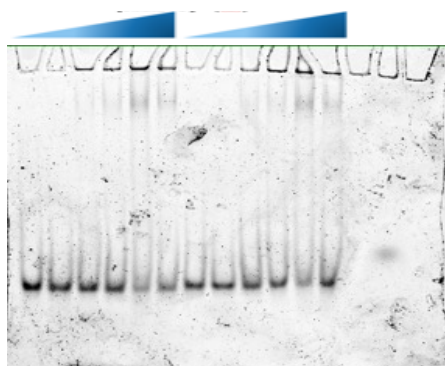

chr3:8596782-8596800

[NKX2-5] (nM)

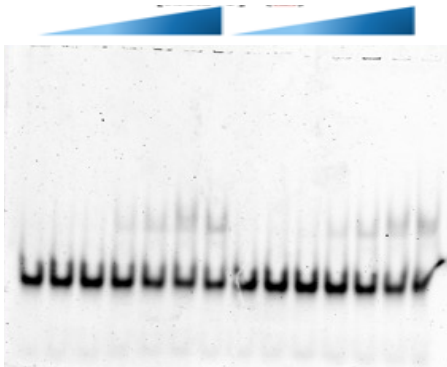

chr1:57623182-57623200

TBX5 (μL)

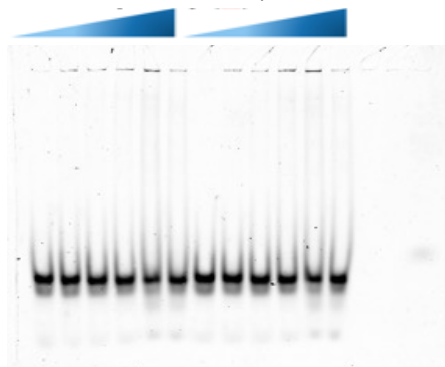

chr7:101950814-101950832

[NKX2-5] (nM)

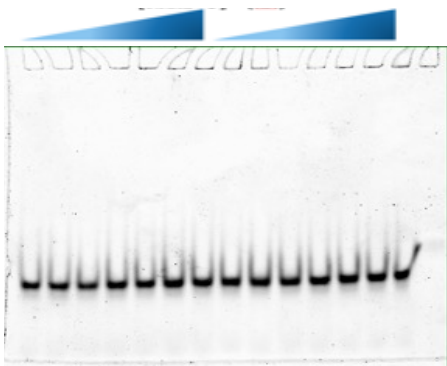

chr4:119047319-119047337

TBX5 (μL)

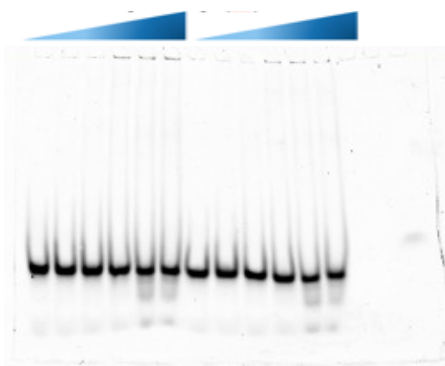

Supplement: Supplementary Figure S2 — EMSA analysis of heart footprint and enhancers for NKX2-5 (left) and TBX5 (right). All EMSA were performed and triplicates. EMSA, electrophoretic mobility shift assay. [file mmc3.pdf]

rs59310144  
[NKX2-5] (nM)

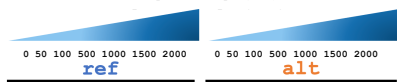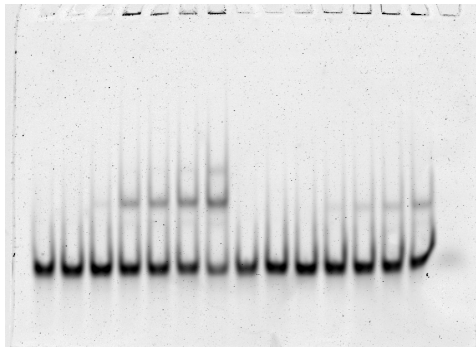

rs76122445  
[TBX5] (nM)

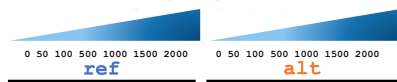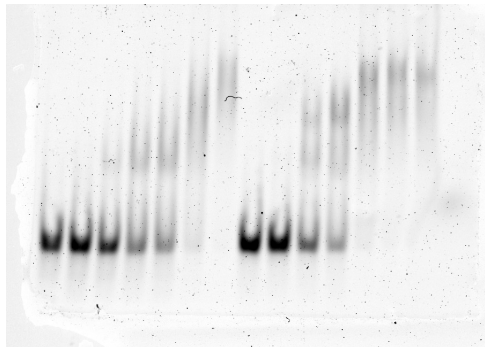

rs6715570  
[NKX2-5] (nM)

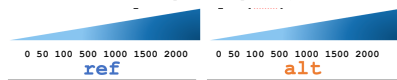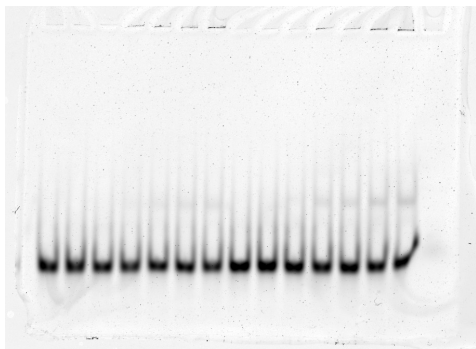

rs7790964  
[TBX5] (nM)

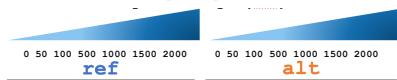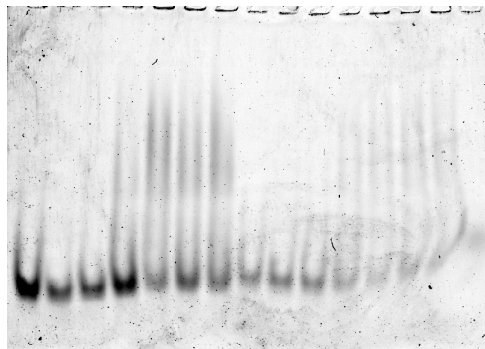

rs61872084  
[NKX2-5] (nM)

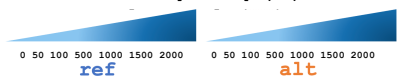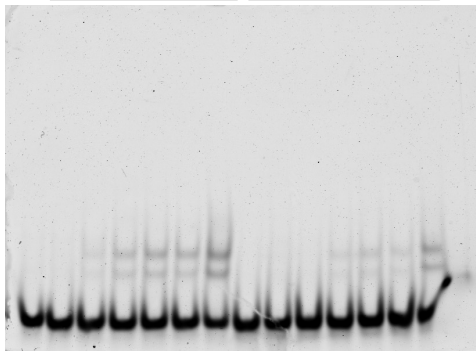

Supplement: Supplementary Figure S3 — EMSA analysis of five CVD-associated SNPs. Figure 3B was derived from EMSA gel of rs59310144 (top left). EMSA, electrophoretic mobility shift assay; CVD, cardiovascular disease; SNP, single nucleotide polymorphism. [file mmc4.pdf]
