# Supplementary material for: Prioritizing cardiovascular disease-associated variants altering NKX2-5 and TBX5 binding through an integrative computational approach
Source: J Biol Chem. 2023 Nov 4;299(12):105423. doi: 10.1016/j.jbc.2023.105423 (PMC10750078; doi:10.1016/j.jbc.2023.105423)
Supplement: Supplemental Figure S4 legend [file mmc5.docx]

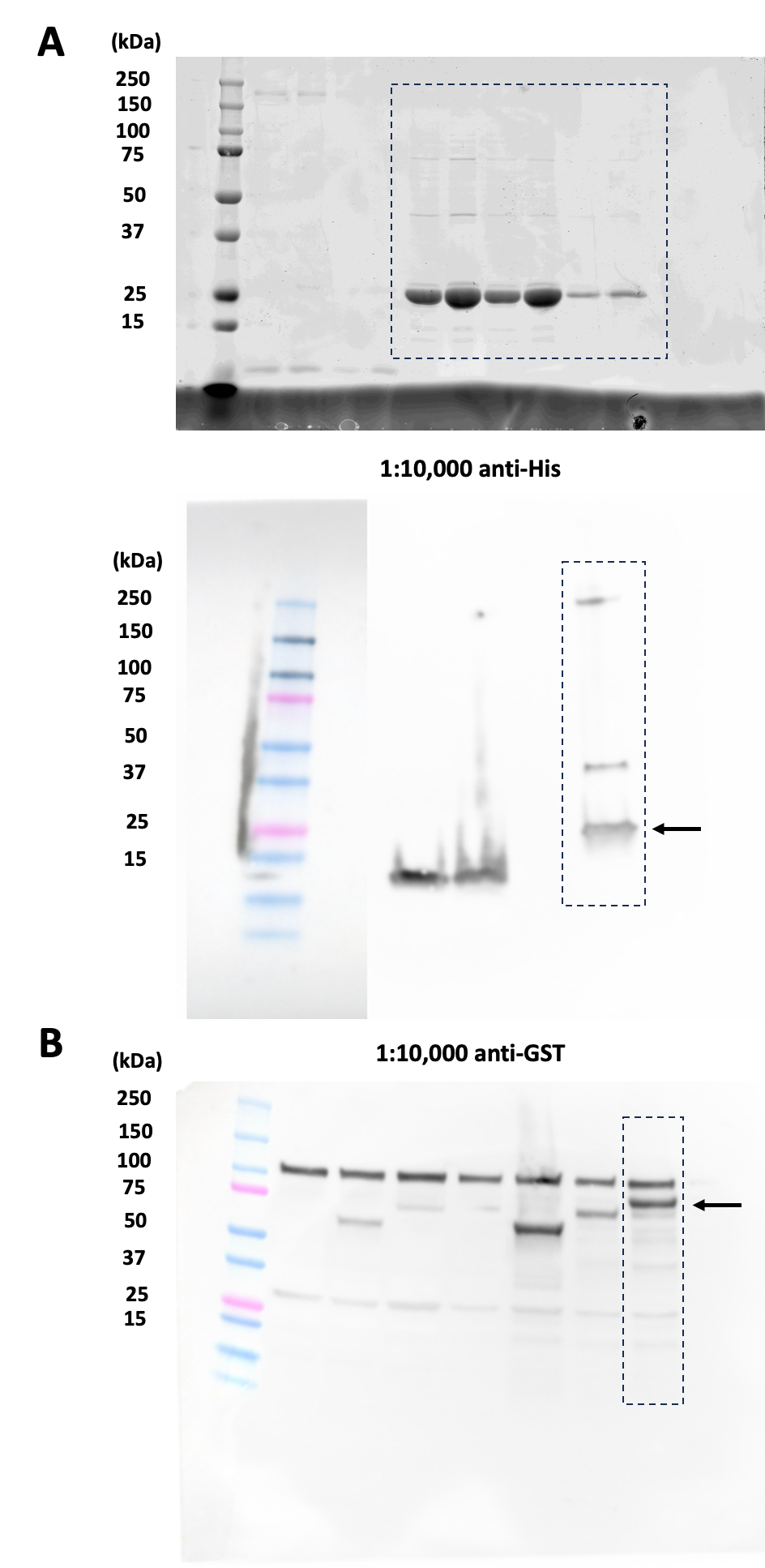


***Supplementary Figure 4:*** *Expression of Full-length TBX5 and T-box domain.* ***A)*** *SDS-PAGE (top) and western blot (bottom) of T-box (26.2 kDa) after Ni-NTA purification.* ***B)*** *Western blot of cell-free expressed full-length TBX5 (85 kDa). The dashed line represents wells of TBX5 and T-box domain used in this work. Specific western blot bands for TBX5 and the T-box domain are pointed with an arrow.*
