# Supplementary material for: Prioritizing cardiovascular disease-associated variants altering NKX2-5 and TBX5 binding through an integrative computational approach
Source: J Biol Chem. 2023 Nov 4;299(12):105423. doi: 10.1016/j.jbc.2023.105423 (PMC10750078; doi:10.1016/j.jbc.2023.105423)
Supplement: Supplementary Tables — Supplementary Table 1: Oligonucleotides used in this work. Supplementary Table 2: CVD-associated SNPs with differential gene expression in cardiac tissue and predicted impact on NKX2-5 DNA binding. [file mmc6.docx]

| **Name** | **Sequence** |
| --- | --- |
| NKX2-5 enhancer  chr22:25120040-25120058 | 5'- ACTTCTTGAGTGCCTGCTCGTGCCAATGCCGCCGTAAG -3' |
| NKX2-5 enhancer  chr3:8596782-8596800 | 5'- CCATGCTATCATCACTCACGTGCCAATGCCGCCGTAAG -3' |
| NKX2-5 enhancer  chr7:101950814-101950832 | 5'- AGTGCTGGGATTACAGGCCGTGCCAATGCCGCCGTAAG -3' |
| TBX5 enhancer  chr2:30359836-30359854 | 5'- TGGTGCTGACAGCTGGGACGTGCCAATGCCGCCGTAAG -3' |
| TBX5 enhancer  chr1:57623182-57623200 | 5'- GCCGGCAGAGCTGACAGGCGTGCCAATGCCGCCGTAAG -3' |
| TBX5 enhancer  chr4:119047319-119047337 | 5'- AAGTGCTGGGATTACAGGCGTGCCAATGCCGCCGTAAG -3' |
| rs6715570-ref | 5'- TAACACTCATGAAAATGTCTCGTGCCAATGCCGCCGTAAG -3' |
| rs6715570-alt | 5'- TAACACTCAAGAAAATGTCTCGTGCCAATGCCGCCGTAAG -3' |
| rs61872084-ref | 5'- AATTCAACACTTTCATTAAACGTGCCAATGCCGCCGTAAG-3' |
| rs61872084-alt | 5'- AATTCAACATTTTCATTAAACGTGCCAATGCCGCCGTAAG -3' |
| rs59310144-ref | 5'- AGAAGTCAAGTAATCTGTCACGTGCCAATGCCGCCGTAAG -3' |
| rs59310144-alt | 5'- AGAAGTCAAATAATCTGTCACGTGCCAATGCCGCCGTAAG -3' |
| rs7612445-ref | 5'- TTAAGAGAGGGTCAATGATACGTGCCAATGCCGCCGTAAG-3' |
| rs7612445-alt | 5'- TTAAGAGAGTGTCAATGATACGTGCCAATGCCGCCGTAAG-3' |
| rs7790964-ref | 5'- AGGACTTCACAGACACTACTCGTGCCAATGCCGCCGTAAG-3' |
| rs7790964-alt | 5'- AGGACTTCATAGACACTACTCGTGCCAATGCCGCCGTAAG-3' |
| IR 700 Primer | 5’- /5IRD700/CTTACGGCGGCATTGGCACG -3’ |
| TBX5 Cloning pEU Forward | 5'- CTGTATTTTCAGGGCATGGCCGACGCAGAC -3’ |
| TBX5 Cloning pEU Reverse | 5'- CGTAAATTCTATACAACTACAAGCTATTGTCGC -3’ |

**Supplementary Table 1**

**Supplementary Table 2**

| **Query SNP** | **LD SNP** | **Chr** | **Position** | **Ref score** | **Alt score** | **deltaSVM Score** | **Gene** | **Tissue** | **P value** | **Association to CVD** |
| --- | --- | --- | --- | --- | --- | --- | --- | --- | --- | --- |
| rs17074987 | rs59310144 | 13 | 50917644 | G  (0.56) | A  (0.06) | -0.5 | *RNASEH2B* | Heart - Atrial Appendage | 2.33E-39 | Identified as differentially expressed in CVD risk events |
| rs6435862 | rs6715570 | 2 | 214808716 | T  (0.217) | C  (0.92) | 0.7 | *BARD1* | Heart - Atrial Appendage | 2.39E-25 | Associated with developmental delay and coarctation of aorta in early organogenesis and heart development |
| rs11245347 | rs61872084 | 10 | 124750493 | C  (0.44) | T  (-0.2) | -0.63 | *METTL10* | Heart - Left Ventricle | 6.81E-27 | A high concentration of enzymatic product (tHcy) is correlated with kidney dysfunction and CVD |
| rs57812663 | rs7790964 | 7 | 35260273 | C  (0.52) | T  (-0.13) | -0.66 | *TBX20* | Heart - Atrial Appendage | 1.15E-12 | Essential for proper heart development. |
| rs7612445 | rs7612445 | 3 | 179455191 | G  (-0.19) | T  (0.52) | 0.72 | *GNB4* | Heart - Atrial Appendage | 2.01E-70 | Associated with regulation of heart rate stability and identified in patients with higher cardiovascular mortality risk |
